# *Pseudomonas aeruginosa* Swarmer Cells Adaptation Toward UVc Radiations

**DOI:** 10.3389/fmicb.2019.00556

**Published:** 2019-04-02

**Authors:** Salma Kloula Ben Ghorbal, Kalthoum Chourabi, Lobna Maalej, Aouatef Ben Ammar, Hadda-Imene Ouzari, Abdenaceur Hassen, Habib Jaafoura, Abdelwaheb Chatti

**Affiliations:** ^1^Laboratoire de Traitement des Rejets Hydriques, Centre des Recherches et des Technologies des Eaux, Technopôle Borj Cedria, Nabeul, Tunisia; ^2^Service Commun de Microscopie Électronique à Transmission, Faculté de Médecine de Tunis, University Tunis El Manar, Tunis, Tunisia; ^3^Laboratoire des Microorganismes et Biomolécules Actives, Faculté des Sciences de Tunis, Physiques et Naturelles de Tunis, University Tunis El Manar, Tunis, Tunisia

**Keywords:** *Pseudomonas aeruginosa*, UVc, swarming, fatty acids, membrane, ultrastructure

## Abstract

Swarming is the most rapid surface motility allowing *Pseudomonas aeruginosa* bacteria to rapidly colonize new surfaces. However, swarming behavior is affected by environmental factors like ultraviolet irradiation (UVc). UVc radiation is the most disinfection technology usually applied for wastewater and proven to be effective to inactivate microorganisms. However, efficiency against motile bacteria is not yet studied. This study aims to explain the mechanisms of resistance of swarmer *P. aeruginosa* cells toward UVc exposure. *P. aeruginosa* liquid cultures were allowed to swarm across a semisolid surface for 18 h and directly exposed to UVc radiations. Emergent swarmer colonies, revealed after re-incubation, were selected to study biofilm formation, fatty acid (FA) composition, and ultrastructure. Our results showed that membrane adaptation to UVc radiations was seen in *Pseudomonas* cells by an increase of cyclic fatty acid (CFA) content, confirming the role of cyclopropane in radio-resistance of swarmer cells. Furthermore, electron microscopic study confirmed that over production of S-layer is believed to be a protective form adopted by *P. aeruginosa* swarmer cells to resist after 5 min of UVc exposure. Moreover, membrane disintegration is the lethal effect observed after 15 min of UVc exposure. In the other hand, study of biofilm production showed an enhancement of biofilm formation, of swarmer cells mainly after 15 min of UVc exposure. There results confirmed that swarming process is highly correlated with particular FA composition of *P. aeruginosa* membrane and that radio-resistance of swarmer cells is highly supported by CFA biosynthesis and S-layer overproduction.

## Introduction

*Pseudomonas aeruginosa* is adept at coordinating individual cells to contribute in some surface-associated behaviors. This ability provides more protection to *Pseudomonas* from environmental aggression and improves access to nutrients (Merritt et al., 2007).

*Pseudomonas* possesses three types of movement, namely, swimming, twitching, and swarming. These movements depend on medium viscosity. Swarming motility involves synchronized and fast movement of a bacterial population across a semisolid surface (Fraser and Hughes, 1999). This movement is frequently typified by a dendritic colonial appearance. Previous studies revealed that swarming of *P. aeruginosa* depends on both flagella and type IV pili, which mediate this movement (Overhage, 2008). Moreover, swarming movement depends on rhamnolipid production. Rhamnolipids are supposed to enable swarming cells to surmount the surface tension of the water, in swarming media (Overhage, 2008). This form of motility is an early state in biofilm formation and is consequently extremely important in the structuring of the biofilm. The linkage between biofilm formation and motility tends to be multifaceted because both processes involve similar components.

Biofilms are complex structures in which bacterial populations form a matrix. Cells in biofilms grow in communities, when nutriments are accessible. Studies of *P. aeruginosa* biofilms have shown that cells in biofilm and planktonic cells are phenotypically different (Elasri and Miller, 1999). *In vitro*, biofilm formation follows several steps: beginning by surface adhesion of bacteria, followed by micro colony development by colonial motility and a later arrangement of a heterogeneous, ordered biofilms characterized by cell aggregates and channels witch separate them.

Furthermore, previous research has shown that rhamnolipids are important in biofilm structure, through maintaining channels for the clearance of waste and metabolic intermediates or through determining the multidimensional growth of the biofilm (Elasri and Miller, 1999).

In addition, the bacterial membrane plays an important role in helping bacteria to adapt to stressful conditions. The change in lipid composition and especially in fatty acids (FAs) composition enables bacteria to preserve membrane functions in the face of stressful conditions. The relationship between change in lipid composition and ultraviolet irradiation (UVc) exposure was well established (Kloula et al., 2013; Maâlej et al., 2014; Khefacha et al., 2015).

To control the spread of this pathogen, several chemical substances, like chlorine and chloramines, have been used. However, by the use of these antibacterial, harmful products are generated. Therefore, UVc irradiation is successfully used, as an alternative to chemicals. The UVc radiation remains the most effective technology for germicidal purposes. It is lethal to microorganisms including bacteria, spores, viruses, yeast, and algae (Khefacha et al., 2015).

In the present study, the purpose is to understand the nature of the complex adaptation of swarmer cells to UVc radiations by study of biofilm formation, rhamnolipid production, membrane FA composition and changes in cell morphology, of *P. aeruginosa* swarmer cells.

## Materials and Methods

### Bacterial Strain and Growth Conditions

*Pseudomonas aeruginosa* PAO1 was supplied by Pasteur Institute (Tunisia). *P. aeruginosa* was routinely cultivated on Tryptic soy Broth (TSB) at 37°C during 24 h. One percent of the overnight culture was then added to Trypto casein Soya Agar (TSA) and grown until the desired optical density (OD) was achieved.

### Swarming Assay

Media used for assay consisted of 0.6% (wt/vol) (Biolife)–agar with 30 g/l TSB, to which 5 g/l glucose was added. For swarming assay, liquid cultures of *P. aeruginosa* were grown overnight in 10 ml of TSB at 37°C, without shaking. After incubation, the OD is adjusted to 0.6 at 570 nm (Spectro UV–vis Dual Beam, UVS-2700, Labomed, Inc., Los Angeles, CA, United States) Then, 10 μl of the liquid culture served to inoculate semisolid media. Dried swarming medium was inoculated centrally onto the surface of the semisolid medium. The colony is first allowed to grow for 18 h at 37°C. The swarming distance was calculated and photographed (Liaw et al., 2004).

### UVc Treatment of *P. aeruginosa* Swarmer Cells

#### Exposure Chamber of UVc Treatment

The laboratory UV device was built with the cooperation of the company Guy Daric S.A (Aubervilliers, France). This prototype contained an irradiation board with six Petri dishes locations. A germicidal low-pressure mercury vapor discharge lamp (UV emission at 254 nm) could be adjusted to ensure homogenous exposure. The lamp was supplied via electric ballast. An extractor was used to remove the ozone produced in the irradiation room. A digital radiometer allows us to measure the UV radiation intensity (I: 4 mW/cm^2^) (Vilbert–Lourmat). The UVc doses are calculated as the product of radiation intensity (I = mW cm^-2^) and the exposure time (t = s). Based on our previous study (Kloula et al., 2013), toxic effect was shown for six UVc doses and inactivation graphs were constructed by plotting the logarithm of the surviving fractions of bacteria log (N/N0) against UVc doses. In the other hand, swarming preliminary assay has orientated the choice of the UVc doses for the present study. Only two UVc doses were retained corresponding to 5 and 15 min of exposure to UVc radiations. Indeed, we revealed emergence of colonies after UVc treatment, thing not seen for all other tested UVc doses.

#### Emergence of *Pseudomonas aeruginosa* Colonies in the Swarm Zone, After UVc Exposure

The colonies allowed to grow for 18 h were exposed, separately to UVc radiations for 5 and 15 min and then re-incubated for 18 h, under the same conditions. In parallel, untreated colony was allowed to grow on semi solid media for 36 h, without being exposed to UVc rays. After 5 min of exposure, a thin layer of plentiful colonies marked the outline of the swarming zone. These colonies of the contour were scarped and studied for their aptitude to form biofilm. Their FA composition was determined. Emergent colonies in the swarmer zone were revealed, after 15 min of exposure. These colonies represent an interesting population to be collected for all the subsequent analysis. For untreated plates, colonies were taken from the edge of the swarm zone.

### Biofilm Assay

#### Congo Red Agar (CRA) Method

The Congo red agar (CRA) assay was done according to the protocol of Freeman et al. (1989). The medium used is composed by brain heart infusion broth 37 g/l, sucrose 0.8 g/l, agar–agar 10 g/l, and Congo red stain 0.8 g/l. Plates were directly inoculated with swarmer picked colonies: emergent colonies of 5 min treated plate and colonies of the contour of 15 min treated plate. CRA plates were incubated aerobically for 24 h at 37°C. According to Freeman et al. (1989), biofilm producer colonies are black colored while biofilm negative strains are pink colored.

#### Microtiter Plate Method (MTP)

Polystyrene microtiter plate served to perform biofilm assays (O’Toole and Kolter, 1998). The bacterial swarmer cells collected from the swarming plates, as previously explained, were resuspended in TSB, until an OD600 of 0.6 was achieved. Wells were inoculated with 200 μl of *P. aeruginosa* cultures. After inoculation, plates were incubated at 37°C during 48 h. Biofilm was quantified by staining the biofilm-containing pegs on the lid with 1% (wt/vol) crystal violet solution for 15 min. To eliminate un-bound dye in PBS solution, we carefully proceed to washing step. Then, we measure absorbance at 595 nm using a plate reader spectrophotometer (BioRAD i Mark^TM^) which provide an indirect measure of the biomass attached to each peg (O’Toole and Kolter, 1998).

### Rhamnolipids Production

The most common used method for qualitative screening of rhamnolipids is the cetyltrimethylammonium bromide (CTAB) agar test. Trypto Soy agar (TSA) plates containing 0.02% CTAB and 0.0005% methylene blue (l%) were inoculated with 2 ml of an overnight LB culture of *Pseudomonas* swarmer cells, scarped from the different areas of the swarming plates, as it was explained in the CRA section method. The lucid zone neighboring the bacterial spots was visualized, after an overnight incubation at 37°C. This zone is a proof of rhamnolipid production (Siegmund and Wagner, 1991).

### Extraction of Total Lipids

Fatty acid composition of colonies scarped from the contour of the swarming zone of 5 min treated plates and emergent colonies from 15 min treated plates was examined. The lipid extraction procedure was carried out according to the protocol of Bligh and Dyer (1959). A monophasic system (dichloromethane/ methanol; 3:1 v/v) was used to realize extraction. Then, chloroform layer obtained after centrifugation was removed. A rotary evaporator at 40°C served to evaporate chloroform layer. Hexane was used to dissolve lipid residue. Finally, storage of lipid extract was carried out until chromatographic analyses, under a nitrogen atmosphere (Bligh and Dyer, 1959).

### Fatty Acids Analysis

The membrane FA composition was determined on a GC system [Agilent Technologies 6890NR model (Network GC System)]. This system was equipped with a flame ionization detector (FID) and an electronic pressure control (EPC) injector. A polyethylene glycol fused silica capillary column (Innowax, 30 m × 0.25 mm × 0.25 μm film thickness) purchased from Agilent (Wilmington, DE, United States) was used. The column was operated at 150°C for 1 min; the temperature was raised by 15°C/min^-1^ to 210°C for 5 min and then raised by 5°C min^-1^ to 250°C and maintained until the end of analysis during 25 min. N_2_ was used as the carrier gas at a flow rate of 150 kPa and H_2_ at a flow rate of 25 ml/min (Aloui et al., 2010). Peak areas were calculated using chromatography software (Agilent Technologies ChemStation FamilyR data analysis) (Kloula et al., 2013). Known standards were injected and their retention times were compared to those of the FAMEs. Relative percentages of each FA were calculated.

### Electron Microscopy

Studied samples were collected from different zones of swarm bacterial colonization, previously explained in the CRA section method. After the supernatant was discarded (10,000 g for 5 min at 4°C), the *P. aeruginosa* pellet was prefixed with 3% glutaraldehyde in 0.2 M sodium cacodylate buffer (pH 7.4). Samples were postfixed for 2 h with 1% osmium tetroxide (v/v) in 0.1 M sodium phosphate buffer (pH 7.4). After fixation, the sample was centrifuged at 1800 *g* for 2 min, the pellet washed three times in 0.2 M Na-cacodylate buffer (pH 7.4). Then, graded series of ethanol served to dehydrate treated samples. The TEMs were taken with a jeol-1010 electron microscope operating at 80 kV.

### Statistical Analysis

Average values of triplicates were given, and the deviation was less than 5% of each value (the mean value ± the standard deviation of replicate value). Significance was assessed using the Student’s *t*-test.

## Results

### Sensitivity of *P. aeruginosa* Swarmer Cells to UVc Rays

**FIGURE 1:**
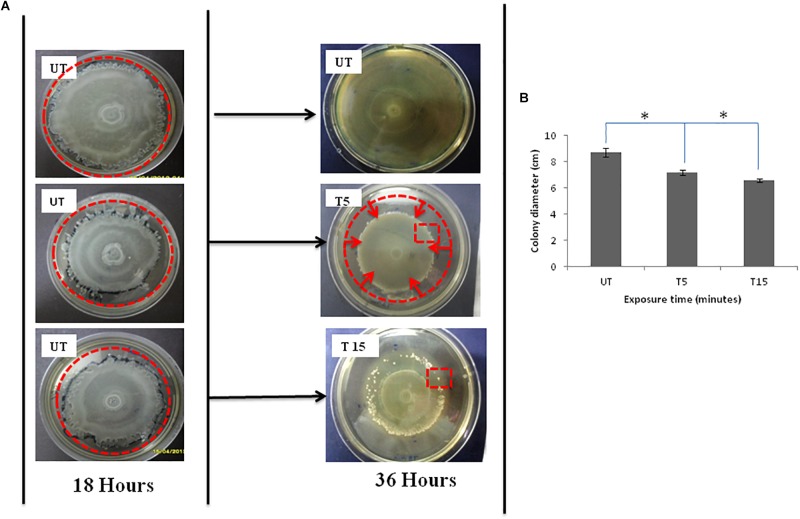
**(A)** Photographs of Petri dishes showing the effects of UVc exposure on swarmer cells of *P. aeruginosa* for the case of (T5) 5 min of treatment and (T15) 15 min of treatment. Note that (UT) represents Petri dishes of untreated samples. The UVc radiation of strength I = 4 mW/cm^2^ is switched on after the colony was allowed to grow for time *t* = 18 h. After the UVc radiation is switched off, the bacterial colony was allowed to recover overnight (*t* = 36 h). Plates were photographed against a black background so that zones of bacterial colonization appear white and uncolonized agar appears black. The zone of migration is marked by a disrupted line red circle in (UT) photograph, the zone of narrowing is also marked by a disrupted line red circle in (T5), and the red square in T15 shows some emergent colonies in the zone of regression. **(B)** Colony diameter of swarming colonies before (UT) and after exposure to UVc radiations for 5 (T5) and 15 (T15) min. (^∗^) shows significant differences.

*Pseudomonas aeruginosa* swarmer cells move quickly from the colony center as multicellular rafts. Then, they pause and undertake several dedifferentiations. Regular cycles of migration and consolidation form a colony characterized by a pattern of concentric rings (Figure 1A-18 h UT), with a diameter of 8.7 cm (Figure 1B-UT). In the monolayer, cell density is elevated and generally homogeneous throughout the swarm, rising slightly in the edge. Then, the colony grows into a featureless mat, when the borders of the plate are reached by the monolayer (Figure 1A-36 h UT).

After exposure to incremental UVc doses and re-incubation, the colony is not observed to simply stop after radiation but morphology change was also observed (Figure 1A-T5 and T15). After 5 min of exposure, the diameter of the migration area is 7.2 ± 0.05 cm with a significant (*P* < 0.05) regression of 1.5 cm, compared to untreated colony (Figure 1B-T5). All of bacteria are observed to reach the limits of the colony. However, this movement does not help swarmer cells to evade the UVc radiation. The front side of the migration forms a noticeable halo more contracted than that observed before exposure (Figure 1A-T5). For prolonged exposure time (15 min), we noticed a narrowing of the migration surface accompanied with the emergence of multiple resistant colonies (Figure 1A-T15). Therefore, the diameter decreased significantly (*P* < 0.05) from 8.7 ± 0.03 to 6.6 ± 0.01 cm (Figure 1B-T15).

After 5 min of exposure, colonies from the halo and emergent colonies, after 15 min of exposure were scarped and examined for their aptitude to produce rhamnolipid and to form biofilm on glass surface, on CRA and in microtiter plates.

### Biofilm Implication and Rhamnolipids Production

**FIGURE 2:**
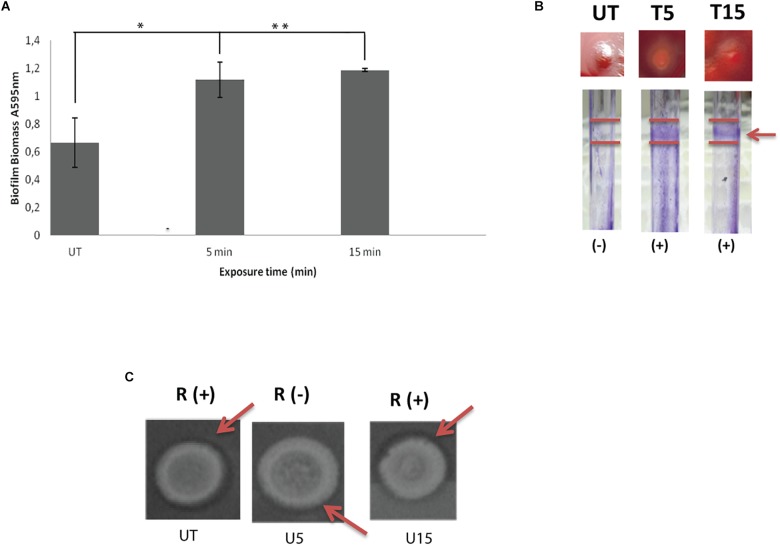
Influence of UVc treatment on biofilm biomass **(A)**, CRA binding and glass tubes assay **(B)**, and rhamnolipid production **(C)**. **(A)** Results are shown for biofilm formation (A595 nm of crystal violet as an indirect measure of biofilm biomass). Biofilms were grown for 48 h at 37°C. UT: untreated bacteria, T5: 5 min of UVc treatment, T15min: 15 min of UVc treatment. Error bars represent the standard errors of the means for a representative assay performed in triplicate. (^∗^) shows significant difference (*P* < 0.05) for each exposure time and (^∗∗^) shows no significant difference. **(B)** In wells photographs, (-) indicates lack of pellicles and (+) indicates presence of pellicles. The arrow shows the pellicle. **(C)** In rhamnolipid test, a dark halo surrounding the colonies on the test plate indicates rhamnolipid production. R (+) indicates positive rhamnolipid production and R (-) indicates a reduced rhamnolipid production. Arrows show the halo surrounding the colony.

The response profile including ability of untreated swarmer cells and UVc exposed swarmer cells to produce biofilm was marked by a dose-dependent response. As seen in Figure 2A, biofilm yields of *P. aeruginosa* improved significantly (*P* < 0.05), when swarmer cells are pre-exposed to UVc radiations for 5 min. Moreover, *P. aeruginosa* showed a progressive enhancement in biofilm formation in pegs with about twofold more biomass after 15 min of exposure, compared to untreated swarmer cells. These changes are concomitant to biofilm formation on glass surface (Figure 2B). Biofilm production tested on glass surface has demonstrated that biofilm formation was considered as a ring of cells adhered to the glass wall at the air-liquid interface. Our results showed that untreated *P. aeruginosa* swarmer cells were unable to produce biofilm on the air–liquid interface of glass tubes. Interestingly, we noted the presence of this ring for swarmer cells pre-exposed to UVc rays during 5 and 15 min (Figure 2B).

On CRA, representative phenotypes of the three morphotypes are shown in Figure 2B. Untreated colonies appeared like stronger mucoid morphotype. After 5 min of treatment, colonies appeared white on blood agar and accumulated the dye Congo red in the center of the colony. As known, Congo red attaches extracellular matrix components and is taken as a surrogate marker for biofilm production. After 15 min of treatment, colonies are highly mucoid resembling untreated colonies (Figure 2B).

Concerning rhamnolipid production, the RL-producing strains are revealed by a dark halo around the colony, allowing easy identification of the presence of RLs. Before UVc treatment, the dark halo is visible showing an interesting rhamnolipid production. However, rhamnolipid production was inhibited after short exposure of swarmer cells to UVc rays (5 min). Interestingly, rhamnolipid production was enhanced after longer exposure and the dark halo was seen (Figure 2C).

Swarmer cells, pre-treated to UVc radiations, are showing an interesting profile including important production of biofilm on different surfaces and induced rhamnolipid production after 15 min of exposure. Their FA composition was also examined and compared to the FA composition of planktonic cells.

### FA Composition in *Pseudomonas* Planktonic Cells

The comparison of the FA proportions of the total lipids showed an important proportion of both saturated (60%) and cyclic (27%) FAs. The major saturated FAs (SFAs) were hexadecanoic (palmitic) acid (C16:0) and octadecanoic (stearic) acid (C18:0) and the major cyclic FA was the cis-9, 10-methylenehexadecanoic acid (cyc17; 26%). Two monounsaturated FAs (MUFAs): hexadecanoic (palmitoleic) acid (C16:1ω7) and octadecanoic (oleic) acid (C18:1ω9) were identified (Table 1).

**Table 1:** Fatty acid profiles in planktonic and swarmer cells of *P. aeruginosa* with and without UVc treatment.

	Cell movement				
	
Fatty acids	Planktonic cells				
		
	UT	UT	T5	T15	T30
C14:0	3.46 ± 0.06^e^	**14,26** ± 0.18^a^	**3,69** ± 0.17^b^	**4,13** ± 0.13^c^	**3,64** ± 0.24^d^
C16:0	42.22 ± 0.56^e^	**28,48** ± 0.21^a^	**32,69** ± 0.26^b^	**38,2** ± 0.46^c^	**32,76** ± 0.76^d^
C16:1ω7	5.07 ± 0.07^e^	1,81 ± 0.07^a^	1,89 ± 0.07^a^	3,81 ± 0.07^c^	2,46 ± 0.15^d^
C17:0	2.97 ± 0.019^e^	7,43 ± 0.19^a^	6,28 ± 0.09^b^	5,47 ± 0.09^c^	5,89 ± 0.21^d^
C17cyc	**26.01** ± 0.22^e^	7,75 ± 0.20^a^	6,19 ± 0.11^b^	10,45 ± 0.15^c^	8,08 ± 0.23^a^
C18:0	**10.74** ± 0.1^e^	**35,69** ± 0.32^a^	**41,01** ± 0.78^b^	**31,19** ± 0.46^c^	**40,52** ± 0.75^d^
C18:1ω9	5.94 ± 0.09^e^	3,22 ± 0.16^a^	5,51 ± 0.19^b^	3,7 ± 0.09^c^	4,02 ± 0.07^d^
C19:0	0,48 ± 0.02^a^	0,44 ± 0.03^a^	0,75 ± 0.08^b^	0,94 ± 0.01^c^	1,13 ± 0.09^d^
C19cyc	1 ± 0.01^e^	0,46 ± 0.29^a^	0,92 ± 0.06^b^	1,26 ± 0.07^c^	0,54 ± 0.02^a^
C18:2ω6	0,04 ± 0.02^e^	0,13 ± 0.04^a^	0,69 ± 0.11^b^	0,3 ± 0.01^c^	0,28 ± 0.03^d^
C18:3ω6	0,63 ± 0.12^e^	0,18 ± 0.05^a^	0,1 ± 0.02^b^	0,34 ± 0.02^c^	0,05 ± 0.01^d^
C18:3ω3	1,39 ± 0.18^e^	0,09 ± 0.01^a^	0,21 ± 0.03^b^	0,16 ± 0.06^a^	0,58 ± 0.09^d^
SFA	59,86	86,3	84,42	79,93	83,94
UFA	13,07	5,43	8,4	8,31	7,38
CFA	27,01	8,21	7,11	11,71	8,62
MUFA	11,01	5,03	7,4	7,51	6,48
PUFA	2,06	0,401	1	0,8	0,909
UFA/SFA	0,22	0,06	0,1	0,1	0,09
CFA/UFA	2,07	1,51	0,85	1,41	1,17


*Values are mean of percentage of total fatty acids identified. SFA: saturated fatty acids, UFA: unsaturated fatty acids, CFA: cyclic fatty acids, MUFA: monounsaturated fatty acids, PUFA: polyunsaturated fatty acids, UT: untreated, T5: UVc treated for 5 min, T15: UVc treated for 15 min. Each value is the mean obtained from three separate analyses. The data presented are shown as the mean value and standard deviation of three replicates. Statistical analysis was done using the Student’s t-test (*P* < 0.05). Comparison was done for each fatty acid of untreated and treated cells and of untreated planktonic and untreated swarmer cells. Different letters mean significant difference (*P* < 0.05) for each fatty acid. Same letters mean no significant difference. Numbers in bold represent significant variations*.

### The Increase of the SFAs Proportions Is More Obvious in Swarmer Cells

**FIGURE 3:**
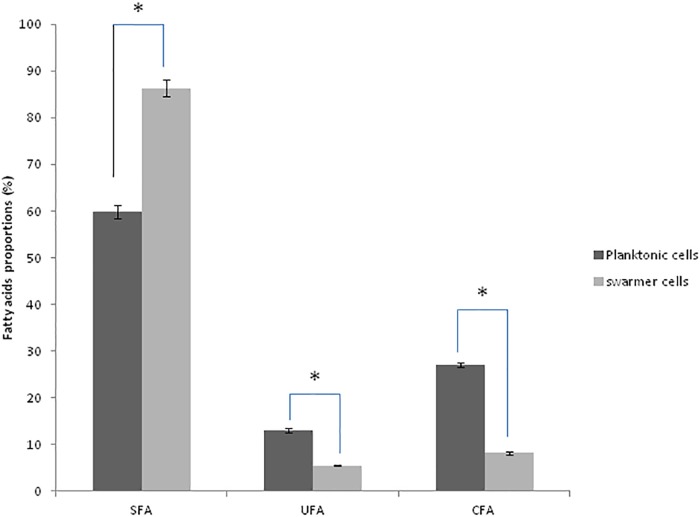
Fatty acid proportions (%) of saturated (SFA), unsaturated (UFA), and cyclic fatty acids (CFA) in planktonic and swarmer cells of *Pseudomonas aeruginosa.* Different letters mean significant difference (*P* < 0.05) for each class of fatty acids. (^∗^) shows significant difference (*P* < 0.05).

To determine whether swarming process in *Pseudomonas* cells affected membrane lipid components, FA composition was determined in planktonic and swarmer cells. First, our results showed that the FA proportions changed when *Pseudomonas* cells undergo swarming process. Our results showed a significant increase in SFA (*P* < 0.05) accompanied by a significant decrease (*P* < 0.05) of both UFAs and cyclic fatty acid (CFA) proportions. In fact, as shown in Figure 3, a significant decrease (*P* < 0.05) of unsaturated fatty acids (UFAs) proportions (from 13.07 to 5.43%) and of CFA proportions (from 27.02 to 8. 21%) was observed, during swarming process. Interestingly, we examined the effect of UVc radiations on the membrane FA composition of swarmer cells.

### Effect of UVc Treatment on the Membrane FA Composition of *Pseudomonas* Swarmer Cells

**FIGURE 4:**
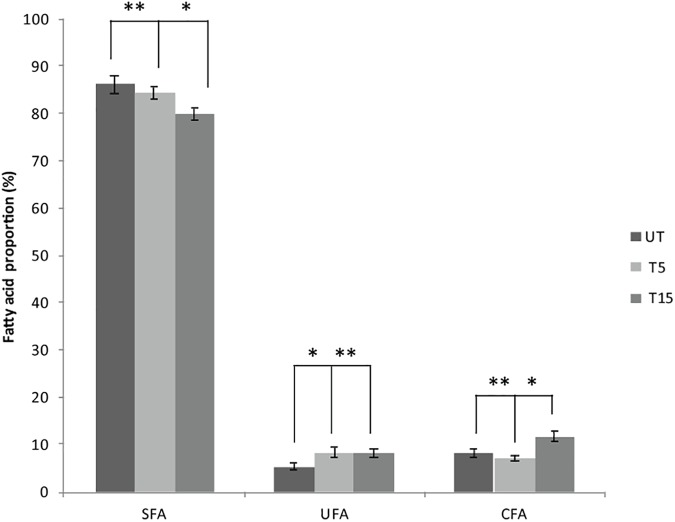
Effect of UVc treatment on the membrane FA composition of *Pseudomonas* swarmer cells. SFA: saturated fatty acids, UFA: unsaturated fatty acids, CFA: syclopropane fatty acids, UT: untreated, T5: 5 min of treatment, T15: 15 min of treatment. (^∗^) shows significant difference (*P* < 0.05) and (^∗∗^) shows no significant difference.

Depending on the cell state, the susceptibility to UVc exposure in planktonic cells is different to that whose take place in swarmer cells. Therefore, in swarmer cells, the shift to unsaturation seemed to take place in a little way, after 5 and 15 min of UVc exposure. SFAs decreased slightly (from 86.3 to 79.93%) and their analogous mono and polyunsaturated (C16:1ω7, C18:1ω9, C18:2ω6, and C18:3) are increasing significantly (*P* < 0.05), in a proportional way (from 5.43 to 8.4%; Figure 4). All of these results were more clearly grouped in Table 1. Furthermore, only a little fraction of UFAs are transformed to CFAs, after 15 min of exposure. In effect, a membrane adaptation was seen in *Pseudomonas* swarmer cells by a significant (*P* < 0.05) increase in CFA content, by means of a significant (*P* < 0.05) rise in cyc17 relative concentrations, mainly after 15 min of exposure (Table 1). After having examined the FA composition of the membrane of swarmer cells, ultrastructure was analyzed by transmission electron microscopy.

### Ultrastructure of *Pseudomonas aeruginosa* Swarmer Cells

**FIGURE 5:**
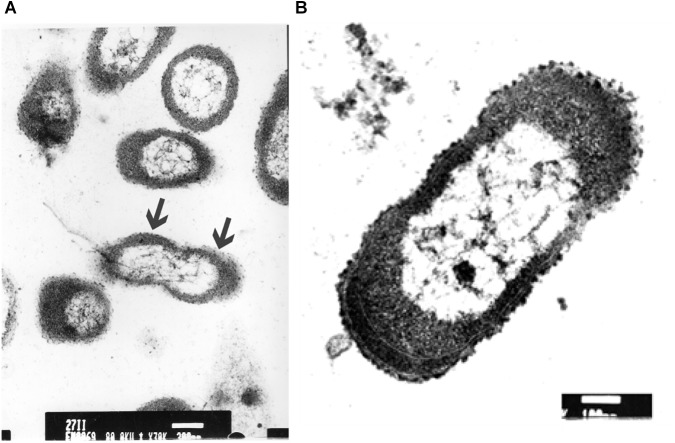
Electron microscopic images of *P. aeruginosa* swarmer cells removed from the swarm edge and observed at the population **(A)** and at the single-cell level **(B)**. Bars: **A** = 300 μm; **B** = 100 μm. In **A**, the arrows show the waved outer membrane with some emanating blebs.

The ultrastructure of *P. aeruginosa* untreated swarmer cells was first carefully analyzed. Cells were either observed at the population (Figure 5A) than the single-cell level (Figure 5B). The general morphology and ultrastructure of the cell envelope of untreated swarmer cells are quite similar to those of Gram-negative rods in general. The untreated control cells possessed a regular cell shape with an intact structure of the inner membrane and a slightly waved outer membrane, with some membrane blebs emanating from cell surface. Untreated swarmer cells showed a homogenous cytoplasm appearance with individual cell membrane structures. The cytoplasm of *P. aeruginosa* swarmer was characterized by the presence of multiples gas vesicles and a single lipid body clearly identified as individual electron-dense dot. As the ultrastructure of swarmer cells was examined before exposure to UVc radiations, changes of swarmer bacterial organization in relation to UVc radiations were easier to be detected.

### UVc Stress Associated Morphological Changes

Transmission electron microscopic (EM) analysis revealed nanometer resolutions to better understand changes of swarmer bacterial organization in relation to UVc radiations after 5 and 15 min of exposure.

**FIGURE 6:**
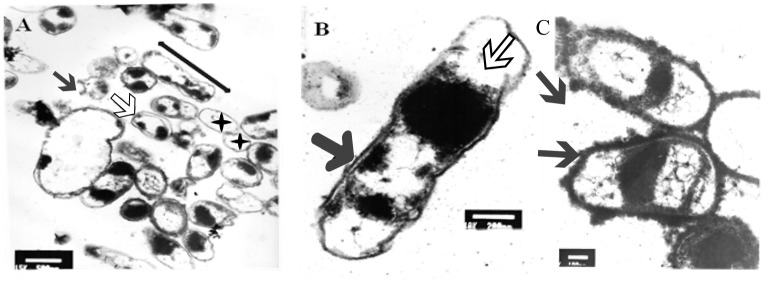
TEM micrograph of a full swarming area profile, after 5 min of exposure, in thin section **(A)** and at higher resolution **(B)**. Electron microscopic images of *P. aeruginosa* swarmer cell with an excess of S-layer at the both ends of the bacterial cell **(C)**. **(A)** Bars = 500 μm; **(B)** bars = 200 μm; **(C)** bars = 300 μm. The micrograph reveals extensive heterogeneity in terms of cell distribution and physiological status. Black star indicates ghost cells with empty envelope structure and damaged cells with condensed cytoplasmic material (black arrowheads) are visible. Double arrow shows cellular dilation in some cells after shorter exposure. Black arrow indicates membrane separation and white arrow indicates early disintegration. Arrows indicate S-layer.

After 5 min of exposure, EM examination of UVc treated cells confirmed the heterogeneous nature of the cellular suspension when compared with untreated cells (Figure 6A). We first noticed the formation of empty and flaccid cells, characterized by an intact cell envelope structure and a loss of intracellular material, in the cell population treated for 5 min (Figure 6A). In the electron microscopy study, the cytoplasm is disorganized and the inner and the outer membrane are separated, the integrity of the outer membrane, however was not maintained in some cases (Figure 6B). In particular, the bacterial envelope was entirely covered with an S-layer. However, under UVc stress an excess of the S-layer protein accumulate at the end of the bacterial cell (Figure 6C).

**FIGURE 7:**
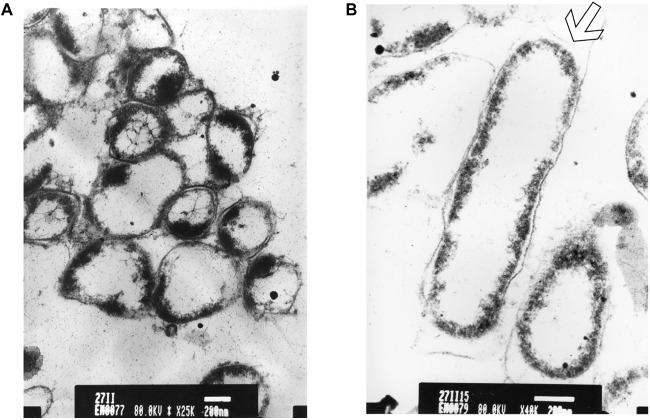
TEM micrograph of a full swarming area profile in thin section after 15 min of exposure **(A)** and at higher resolution **(B)**. **(A)** Bars = 500 μm; **(B)** bars = 200 μm. This section reveals damaged cells with condensed cytoplasmic material (black dots) are visible. The arrow shows cytoplasm shrinkage in the cell pole.

After longer exposure time (15 min), a lethal effect was observed from a cell morphological point of view. With an increase of the treatment time, an elevated antimicrobial effect related to structural differences in the cell wall and cytoplasm of treated cells could be demonstrated (Figure 7A). The cells were essentially characterized by a cytoplasm reduction and expulsion of the cellular content. Moreover, clear fibrous structures in extracellular space may indicate a disruption in cell membrane components that are partially folded (Figure 7B).

## Discussion

Microbiological studies have led to the theory that *P. aeruginosa* can adapt its existence to various different conditions through the upregulation of a great panel of its functional genes.

In the present study, we demonstrate the first approach to examine *P. aeruginosa* swarming phenotype, which reflects life in viscous environments, and present evidence of multifaceted adaptation of this bacterium (Overhage, 2008).

Our results showed that *P. aeruginosa* swarmer cells exhibit adaptive resistance to increased times of exposure to UVc radiations. As seen in the present experiment, after exposure of swarmer cells to UVc radiation, the population starts to accumulate near the edge. Moreover, the external front continues to develop slowly, mainly after 5 min of exposure, because of slow bacterial growth. However, after longer exposure times (15 min) of UVc irradiation, colonies of *P. aeruginosa* were observed to show a narrowing of the migration surface accompanied with the emergence of multiple colonies. A qualitatively similar phenomenon was observed with *Bacillus subtilis* strains exposed to uniform UV radiation (Delprato et al., 2001).

These results obviously support the conclusion that swarming of *P. aeruginosa* is a multifaceted adaptation process in response to viscous surfaces, resulting in a considerable change in swarming phenotype and exhibiting a strong interrelation with biofilm formation. In this context, we studied effect of UVc exposure on biofilm formation to found a correlation between motility and biofilm development. Our results showed that with incremental UVc doses, *P. aeruginosa* cells were able to produce more biofilm in BHI medium. We found a strong correlation between UVc exposure and ability to form biofilm in microtiter wells. So, UVc treated swarmer cells displayed a progressive stronger capacity to adhere to the microtiter plate compared with untreated cells. Accordingly, in this report we have demonstrated that with increased times of exposure to UVc rays, *P. aeruginosa* cells were able to form more important biofilm biomass. The link between motility and biofilm formation is complex because both processes involve analogous components at certain stages and conditions. In addition, the role of swarming movement is not well understood; however, a new report has suggested that swarming behavior may play a role in determining the definitive organization of biofilms (Merritt et al., 2007). It was, also, confirmed that mutants with altered swarming motility were also enable of biofilm development. This may confirm the key role of swarming in early biofilm expansion (Overhage, 2008).

In the other hand, our results have shown that colony morphology was altered after UVc exposure. Therefore, we propose that the diversity of all *P. aeruginosa* morphotypes, revealed at different times of exposure to UVc radiations, could be due to adaptive mutations and selection. Stafford and Hughes (2007) have demonstrated that altered colony morphology on Congo red plates can be linked to loss of curli. On the other hand, many variations in Congo red colony morphology were reported. These variations depend on curli and LPS expression.

Moreover, we investigated whether swarming differences at two UVc exposure times were due to rhamnolipid production, and the data from the staining assays supports that there is a positive correlation between the amount of rhamnolipids produced and the total swarm area covered by *P. aeruginosa*. Previous study has demonstrated that more rhamnolipid production does show a positive correlation with increased swarming behavior.

Potentially, differences in metabolism, genetic regulation, or biomechanics due to changes in environmental conditions could influence the ability of *P. aeruginosa* to tolerate non-ideal environmental conditions and thus would result in observable differences in swarming motility and biofilm formation (Overhage, 2008).

It is known that the inner bacterial cell membrane is responsible of transport, osmoregulation and respiration processes, biosynthesis and cross-linking of peptidoglycan, and synthesis of lipids. Furthermore, there is no doubt that for all these functions membrane integrity is extremely necessary and its disturbance can cause direct or indirect metabolic dysfunction and consequently cell death (Kadurugamuwa and Beveridje, 1995).

Concerning the effects of UVc radiations on the membrane structure and composition, our comparative study of FA composition of planktonic and swarmer cells showed that FA proportions changed when *Pseudomonas* cells undergo swarming process with a significant (*P* < 0.05) increase in the SFA proportion.

In the other hand, alterations in bacterial membrane integrity observed by electron microscopy (EM) can help to explain most of mechanisms leading to cell death after incremental UVc doses. In the current study, membrane structure examined by electron microscopy and membrane composition revealed by Gaz chromatography not only could reveal the direct damage on the bacterial envelope caused by UVc rays, but also demonstrated the link between FAs composition and disintegration of the cell wall. EM study of *P. aeruginosa* swarmer cells showed that, after UVc exposure, the cytoplasm was disordered. Moreover, the inner and the outer membrane were separated. However, the integrity of the outer membrane was not maintained in some cases. Previous studies have revealed the emergence of structures similar to mesosomes in *Staphylococcus aureus*, after treatment with defensins. Moreover, detached cell walls antimicrobial peptides were observed, leading to alteration and permeabilization of the cell wall. Furthermore, incubation of *S. aureus* with antimicrobial peptides resulted in thinning or disintegration of the cell wall, total cell lysis leading to destabilization of the peptidoglycane layer (Shimoda et al., 1995). Additionally, an excess of the S-layer protein was observed at the end of the bacterial cell surface of *P. aeruginosa* under UVc stress condition. Consequently, we suppose that this over production of S-layer may be a protective form adopted by this bacterium to reduce contact with UVc rays. In previous studies, it was confirmed that the S-layer protein has a defensive role for *Lactobacillus acidophilus* ATCC 4356. Earlier, the excess of S-layer was found at the site of separation of the two daughter cells in *Clostridium thermosaccharolyticum*, which prevented the exposure of the newly synthesized parts of the cell wall to the environment (Boot et al., 1996). This excess of S-protein production may ensure whole coverage of the cell wall during cell division (Boot et al., 1996).

Alterations in *P. aeruginosa* cells caused by UVc rays and confirmed with EM were accompanied by a change in lipid composition. All of these mechanisms enable this micro-organism to maintain membrane functions in the face of UVc exposure. The present results confirmed that both UFAs and CFAs proportions were increased insignificantly (*P* > 0.05), after UVc exposure. This increase was mainly balanced by a non-significant decrease (*P* > 0.05) of their analogous SFAs (C14:0 and C17:0 especially). This response is similar to that observed for three serovars of *Salmonella: Salmonella* Typhimurium, *Salmonella* Hadar, and *Salmonella* Zanzibar treated with UVc radiations (Maâlej et al., 2014). Therefore, UFAs are over-produced to scavenge the reactive oxygen species (ROS) and this overproduction is beneficial to ensure more radio-tolerance (Kloula et al., 2013, 2016). The relation between fatty acyl desaturation and bacterial adaptation was reported for a number of strains exposed to heat, cold and oxidative damage (Denich et al., 2003). In addition, our results showed a significant (*P* < 0.05) increase in CFAs proportion, especially after longer exposure time (15 min), concomitant of a significant (*P* < 0.05) decrease in UFAs, supposing a conversion of unsaturated to CFAs. Brown et al. (1997) described an important role of CFA in microbial acid adaptation responses. Furthermore, previous studies proved that the synthesis of CFA in cell membrane phospholipids, during acid adaptation, is an important factor in the protection from several stress conditions (Chang and Cronan, 1999). Recently, study of radio-resistance confirmed the key role of CFA (Kloula et al., 2013; Maâlej et al., 2014). Moreover, the role of CFAs in the radio-resistance is actually confirmed in swarmer cells of *P. aeruginosa*. Duforc et al. (1984) have proved that the presence of a cyclopropane ring in biological membranes stabilize their structure and their dynamic properties.

## Conclusion

Examination of resistant swarmer cells pre-exposed to UVc radiations during 5 and 15 min has demonstrated that this resistance is correlated to a significant increase in CFA content, mainly seen after examination of the membrane FA composition of emergent colonies in 15 min UVc treated plates. This hypothesis about the key role of CFA in radio-resistance could be supported by a change in membrane structure marked by accumulation of S-layer protein at the end of the bacterial cell. These could influence the ability of *P. aeruginosa* to tolerate UVc exposure and thus would result in important ability to form more biofilm biomass in colonized surfaces, after exposure to UVc radiations.

## Author Contributions

SG performed the experiments and wrote the manuscript. KC collaborated in interpretation of TEM micrographs. LM helped in sample preparation and experiment’s designation. HJ and SG assisted with electron microscopy technique. AB conceived and designed TEM samples. H-IO reviewed the manuscript. AH performed UVc prototype. AC conceived and designed the experiments and reviewed the manuscript.

## Conflict of Interest Statement

The authors declare that the research was conducted in the absence of any commercial or financial relationships that could be construed as a potential conflict of interest.
